# Quality of life in coeliac disease: item reduction, scale development and psychometric evaluation of the Coeliac Disease Assessment Questionnaire (CDAQ)

**DOI:** 10.1111/apt.14942

**Published:** 2018-08-20

**Authors:** Helen Crocker, Crispin Jenkinson, Michele Peters

**Affiliations:** ^1^ Nuffield Department of Population Health University of Oxford Oxford UK

## Abstract

**Background:**

A better understanding of coeliac disease can be achieved by assessing health‐related quality of life alongside clinical factors. Existing patient‐reported outcome measures (PROMs) evaluating quality of life in coeliac disease have not been developed in accordance with the US Food and Drug Administration guidelines.

**Aim:**

To develop a PROM in accordance with best practice guidelines, capturing all aspects of quality of life important to adults with coeliac disease.

**Methods:**

Candidate items for the Coeliac Disease Assessment Questionnaire (CDAQ) were refined through item appraisal, expert review, cognitive interviews, and a translatability assessment. A cross‐sectional survey determined further item reduction and the CDAQ's structure. The final CDAQ was administered alongside the Short Form Health Survey Version 2 (SF?36v2) in a second survey to assess construct validity and test‐retest reliability.

**Results:**

Pre‐testing the 64 candidate items revealed a range of issues which guided their refinement and reduction, resulting in the final CDAQ with 32 items representing 5 subscales: stigma (eight items), dietary burden (eight items), symptoms (five items), social isolation (five items), and worries and concerns (six items). Cronbach's alpha ranged between 0.82 and 0.88 for all domains. Further results showed CDAQ scores were more strongly correlated with the SF‐36v2's mental health dimensions, as expected. Intraclass correlation coefficients ranged from 0.79 to 0.89.

**Conclusion:**

The CDAQ is a reliable and valid coeliac‐specific measure that captures all aspects of quality of life important to adults with coeliac disease. Further work is underway to assess the CDAQ's responsiveness to change.

## 1 INTRODUCTION

Coeliac disease is a chronic autoimmune condition affecting approximately 1% of the population.^1^ The immune response is triggered by the consumption of gluten, a protein found in wheat, barley and rye. The only treatment currently available is a gluten‐free diet, which is known to be burdensome, restrictive and challenging in terms of adherence.^2^, ^3^, ^4^ Various aspects of daily life can be affected by following a gluten‐free diet, including travelling, shopping and eating meals outside of the home.^5^

Patient‐reported outcome measures (PROMs) present a unique opportunity to systematically gain insight into patients’ views, which may not overlap with clinical outcomes or biomedical markers.^6^ A broader understanding of the impact of coeliac disease may help to direct care and improve clinical outcomes.^6^ PROMs can also be used as endpoints in clinical trials, which are currently underway to develop and test pharmacological treatment alternatives to a gluten‐free diet.^7^ It is likely that the treatments under development will be supplementary to, rather than a substitute for, the diet.^8^

PROMs that are to be used in clinical trials to support labelling claims should be developed by following the guidance of the US Food and Drug Administration (FDA).^9^ In any case, this guidance is considered best practice for the development of PROMs regardless of their intended use.^10^ The initial steps of development should include qualitative interviews or focus groups with people with the relevant disease to generate candidate items, which then undergo cognitive testing. Once the items have been determined, psychometric properties need to be assessed to evaluate the PROM's quality. Specifically, PROMs must be reliable, valid and responsive to change.^11^

Patient‐reported outcomes in coeliac disease have predominantly been assessed using generic measures, such as the Short Form Health Survey (SF‐36).^12^ However, generic measures are less specific and can be less sensitive than disease‐specific measures. Some coeliac‐specific measures have been developed, for example, the Coeliac Disease Questionnaire (CDQ)^13^ and the Coeliac Disease Quality of Life Survey (CD‐QOL).^14^ A systematic review identified four candidate coeliac‐specific PROMs for use in clinical trials and concluded that none of these meet the standards of the US Food and Drug Administration.^15^ Another systematic review^16^ focused on patient‐reported symptom scores and identified two coeliac disease indices that have been approved by the US Food and Drug Administration. Symptom indices are recommended as end points in clinical trials for new treatments for coeliac disease.^17^ However, they are narrow in their focus and are unlikely to capture all aspects of quality of life that are important to people with coeliac disease. As such, there is a need for PROMs that include other health‐related quality of life domains to capture the broader burden of disease.

Due to the limitations of existing coeliac disease‐specific measures, our aim was to develop a new PROM, capturing all aspects of coeliac‐specific health‐related quality of life, for adults with coeliac disease using best current practice in instrument development.

## 2 METHODS

**Figure 1:**
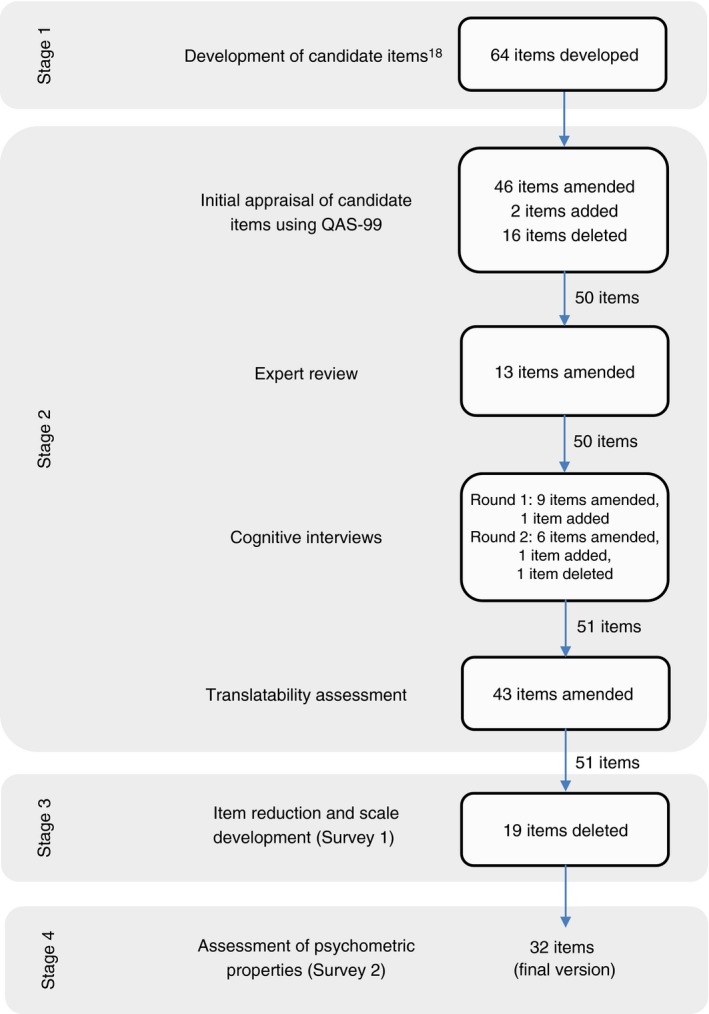
Number of items, and amendments made, at each stage of development of the Coeliac Disease Assessment Questionnaire (CDAQ)

The development of the Coeliac Disease Assessment Questionnaire (CDAQ) was undertaken in four stages (Figure 1). In stage 1, qualitative interviews with 23 adults with coeliac disease informed the development of candidate items and is reported elsewhere.^18^ In stage 2, candidate items were refined following item appraisal, expert review of the items, cognitive interviews and a translatability assessment. The items were amended as necessary after each step of pre‐testing. In stage 3, data collected from a cross‐sectional survey were used to reduce the number of items and identify the CDAQ's dimensions. In the final stage (stage 4), the reliability and validity of the CDAQ was assessed using data from a further cross‐sectional survey. Stages 2 to 4 are reported below, with further details available in the Supporting Information. An item tracking matrix documenting changes made to items during development is available from the corresponding author. Ethics clearance was obtained through the University of Oxford Central University Research Ethics Committee (REC Reference No's: MSD‐IDREC‐C1‐2013‐142; MSD‐IDREC‐C1‐2014‐031; MSD‐IDREC‐C1‐2014‐031).

### 2.1 Stage 2—Refining and pre‐testing the candidate items

#### 2.1.1 Initial item appraisal and expert review

First, candidate items were reviewed using the Question Appraisal System (QAS‐99)^19^ which aids the systematic assessment of questionnaires to identify and resolve common problems (eg, poor question clarity) at an early stage of development. Second, feedback on the retained items was obtained from experts in meetings and interviews in June 2013.

#### 2.1.2 Cognitive interviews

Cognitive interviews were conducted with people with coeliac disease in August 2013 to identify sources of response error within the questionnaire, such as incomprehensible questions.^20^ Detailed notes of any identified problems and suggestions for improving the items were documented and guided item revision. All participants gave written consent.

#### 2.1.3 Translatability assessment

A translatability assessment was conducted to identify and address any cultural or linguistic translatability issues. The assessment was conducted in collaboration with PharmaQuest Ltd (now Corporate Translations, Inc.), a company specialising in the translation and linguistic validation of PROMs. Translators commented on the translatability of the CDAQ's instructions, items and response options, with comments used to further refine the CDAQ.

### 2.2 Stage 3—Item reduction and scale development

Following pre‐testing, a postal survey (Survey 1) was conducted with adult members of Coeliac UK, a UK‐based charity for people with coeliac disease, in September 2014. Eight hundred members meeting the following criteria were invited to participate: aged 18 years or older; self‐reported a medical diagnosis of coeliac disease; and lived in the UK. A random sample, stratified by ethnicity, age and gender, was invited to increase the likelihood of achieving a diverse sample (see Supporting Information for further details).

The survey questionnaire included 51 CDAQ candidate items along with demographic and disease‐related questions (eg, time since diagnosis, adherence to the gluten‐free diet). CDAQ items ask about the past 4 weeks, with all items scored from 1 (“Never”) to 5 (“Always”). No data imputation was undertaken.

#### 2.2.1 Analysis

Data were analysed using IBM SPSS Statistics 20. The aim of the analysis was to determine the structure of the CDAQ and, where necessary, to reduce the number of items.

Prior to principal components analysis, candidate items were considered for removal if more than 5% of data were missing on an individual item; if there was a floor or ceiling effect >50% (ie, at least 50% of respondents selected “never” or “always”); or if inter‐item correlations <0.2 or >0.8 (ie, items were measuring different constructs^21^ or almost the same thing^22^).

Following the removal of candidate items, the Bartlett's Test of Sphericity^23^ and the Kaiser‐Meyer Olkin (KMO) Measure of Sampling Adequacy^24^ were performed to assess the factorability of the data. A significant result for the Bartlett's Test, and KMO values >0.60 indicate the data is factorable.^24^

A principal components analysis with Varimax rotation was used to identify the structure of the constructs. This method was chosen as it tends to produce clearly defined domains^25^ and interpretable solutions.^21^ Components with an eigenvalue greater than one were extracted. During the analysis, items were considered for deletion if they correlated highly with other items (>0.7), indicating item redundancy; or they did not load strongly on any component (all loadings <0.5), indicating a poor fit between the item and components within the measure. Cronbach's alpha was used to assess internal reliability, with values between 0.7 and 0.95 indicating good internal consistency.^26^ Item‐total correlations <0.3^25^ were considered for removal as they contribute little to discriminate between respondents.

A higher order factor analysis was conducted to determine the appropriateness of combining dimension scores to create an overall score.

### 2.3 Stage 4—Assessing the reliability and validity of the CDAQ

Following the reduction in items, a second cross‐sectional postal survey (Survey 2) was conducted with adult members of Coeliac UK. Eight hundred members of Coeliac UK were invited to participate. The same eligibility criteria and sampling strategy as the previous survey (Stage 3) were adopted. The survey questionnaire included the CDAQ, the Short Form Health Survey Version 2 (SF‐36v2),^12^ and demographic and disease‐related questions.

#### 2.3.1 Test‐retest reliability

To evaluate test‐retest reliability, consenting respondents completed a second questionnaire, which included the CDAQ and the following question (“Compared with 2 weeks ago, how would you rate the impact of your coeliac disease on you and your health now?”), rated on a five‐point response scale from “much better” to “much worse”. Respondents reporting that their health was unchanged were included in the analysis. A test‐retest interval of 2 weeks was selected as it is generally considered short enough for no changes to have occurred, but long enough to minimise the risk of respondents recalling their previous answers.

#### 2.3.2 SF‐36v2

The SF‐36v2 is a 36‐item generic measure of health‐related quality of life addressing the eight domains: Physical Functioning, Role‐Physical, Bodily Pain, General Health, Vitality, Social Functioning, Role‐Emotional and Mental Health. In addition, two summary scores can be calculated, the Physical Component Summary and Mental Component Summary. The SF‐36v2 was selected as it is considered to be the leading generic measure,^27^ it has been used in previous studies in coeliac disease, for example,^28^, ^29^, ^30^ and construct validity is commonly assessed against a generic measure.^31^ *T* scores (mean 50, SD 10) are reported, with higher scores indicating better quality of life (original 0‐100 scores are reported in the Supporting Information). *T* scores within 0.3 standard deviations of the mean are considered within the normal range (ie, scores between 47 and 53). QualityMetric Health Outcomes Scoring Software v4.5 was used to calculate SF‐36v2 scores.

#### 2.3.3 Analysis

Internal consistency reliability, test‐retest reliability, and construct validity were evaluated. The internal consistency of each CDAQ dimension was assessed using Cronbach's alpha, with acceptable values ranging between 0.70 and 0.95.^26^ Test‐retest reliability was evaluated using the intraclass correlation coefficient (ICC), with values of 0.70 or above considered acceptable.^26^, ^32^

In terms of construct validity, convergent and divergent validity were assessed by comparing dimensions of the CDAQ and the SF‐36v2. Higher scores on both measures indicate better quality of life, therefore, all correlations were expected to be positive. Overall, the CDAQ subscales and overall index score were expected to correlate more strongly with mental health dimensions and the Mental Component Summary score rather than with the physical health dimensions of the SF‐36v2, with the exception of the symptoms subscale that was expected to correlate more strongly with the physical dimension scores of the SF‐36v2 (expected correlations outlined in full in the Supporting Information).

Based on the literature (eg, 33, 34), it was hypothesised that the CDAQ overall index score would vary by gender, with women expected to report lower scores than men. An independent samples *t* test was used to test this hypothesis. In addition, it was expected that the CDAQ overall index score would discriminate between groups based on self‐reported impact of coeliac disease (scored from 1 “no impact” to 5 “very severe impact”), with higher CDAQ scores for those reporting lower impact. A one‐way ANOVA with Tukey‐Kramer post‐hoc test was used to determine whether the differences between severity groups were significant.

## 3 RESULTS

Candidate items for the CDAQ were refined and pre‐tested, prior to a survey being conducted for further item reduction and identification of the subscales of the CDAQ. Following item reduction, the reliability and validity of the CDAQ was assessed. This process, the number of items and amendments at each stage are shown in Figure 1.

### 3.1 Stage 2—Refining and pre‐testing the candidate items

#### 3.1.1 Initial item appraisal

Using the Question Appraisal System (QAS‐99),^19^ 140 problems were identified across the 64 candidate items. The largest number of problems were due to items lacking clarity (eg, lengthy or awkward wording), being double‐barrelled, or based on potentially problematic assumptions (eg, that respondents have children). Consequently, 46 items were amended, two items were added to address unclear constructs more specifically, and 16 items were deleted as they were conceptually too similar to other items.

#### 3.1.2 Expert review

Nine experts (including clinicians, researchers and Coeliac UK employees) participated in a meeting or interview. Suggestions to improve the candidate items included re‐wording, broadening content (eg, acknowledging the presence of gluten in drinks as well as food), and adding statements to clarify that items should be answered in relation to coeliac disease. Experts also commented on the cultural translatability of some items (eg, “eating out” may not occur in all cultures). Overall, 13 items were amended on the basis of recommendations made by the experts.

#### 3.1.3 Cognitive interviews

Ten people (three men and seven women) with coeliac disease took part in a cognitive interview. Two rounds of interviews were conducted, following which it was deemed that no major issues remained. Participants were aged between 24 and 80 years, the majority of which were White British (n = 9), and had been diagnosed more than 6 years ago (n = 8), and half were married (n = 5) and in full‐time employment (n = 5). Three types of problems were identified: (a) participants answered some questions in general rather than specifically about their coeliac disease; (b) participants interpreted specific words and phrases within the context of items in different ways to each other and (c) participants interpreted items in a different way than intended. Amendments were made to 11 items.

In general, participants felt that the CDAQ comprehensively covered all areas of health‐related quality of life in relation to coeliac disease. While participants accepted the 4‐week recall period, many commented that certain important and potentially problematic experiences, such as having medical tests or going on holiday, were unlikely to have occurred within this time frame.

#### 3.1.4 Translatability assessment

Sixty‐four potential translatability issues were identified across 40 items (out of 51), which were broadly categorised as “cross‐cultural” (n = 27) or “grammatical” (n = 37). The majority of cross‐cultural issues identified arose due to a lack of equivalent vocabulary in the target languages (eg, words such as “condition”) (n = 22), or translations of phrases (eg, “eating out”) where no conceptually equivalent phrases exist in all the target languages (n = 4). One item was potentially difficult to translate due to sociocultural differences, with the concept of following a gluten‐free diet out of personal choice not understood in all cultures. The majority of grammatical issues identified were as a result of inconsistent tenses across items (n = 28), structural errors (eg, missing verbs) (n = 7) and the wording of items not accurately or adequately expressing the underlying concept (n = 2). Sixty‐nine amendments were made to 43 items to address these issues and improve the readability of items.

### 3.2 Stage 3—Item reduction and scale development

A 52% (n = 412) response rate to Survey 1 was achieved. One questionnaire was excluded from the analysis as only the demographics section had been completed. The mean age of respondents was 49.8 years (SD 18.9, range 18‐87), with more women (n = 225, 54.7%) participating than men (n = 186, 45.3%). The majority were White British (n = 348, 84.7%), married or in a civil partnership (n = 270, 65.7%), and in full‐ or part‐time employment (n = 213, 51.8%). The duration of diagnosis of coeliac disease ranged from 1 month to 67 years (mean 8.2 years, SD 10.5).

#### 3.2.1 Item reduction

Fourteen items met the criteria for considering their exclusion. A floor effect was present for eight items and a ceiling effect in one item. Missing data (ie, nonresponse) was minimal, ranging from 0% to 1.2% per item. The correlation matrix revealed a high inter‐item correlation (*r* = 0.82) between items 24 (“felt depressed”) and 36 (“felt down or in low spirits”), suggesting multicollinearity.

Three of these items were retained despite meeting the exclusion criteria. Item 40 (“felt annoyed about the cost of gluten‐free food”) and item 13 (“had nausea or vomiting”) were retained as qualitative interview participants^18^ commented on the importance and severity of these issues. Item 36 (“felt down or in low spirits”) was retained as one but not both items should be removed when a pair of items are highly correlated. Of the remaining 40 items, item 8 (“worried family member could develop coeliac disease”) was the only item with a “not applicable” response option. As this response option was rarely endorsed by respondents (1%, n = 4), the ‘not applicable’ response option was removed.

#### 3.2.2 Subscale development

Bartlett's Test of Sphericity was statistically significant (χ^2^ (780) = 8963.46, *P* < 0.001), and the KMO measure was 0.95. This confirmed that it was appropriate to conduct a principal components analysis.

The principal components analysis (conducted on the 40 retained candidate items) identified six components with an eigenvalue >1, explaining 58.6% of the variance. All items loaded on to at least one component with a loading >0.40. Broadly, the subscales covered stigma (11 items); dietary burden (10 items); worries and concerns (six items); symptoms (six items); social isolation (five items) and future concerns (two items). Six items met the pre‐defined criteria for removal. A further two items were removed due to a poor conceptual fit with other items within its component; and one as it was addressing a similar issue to other items. Removal of these items did not significantly affect the internal consistency of the subscales.

After removing these eight items, a further principal components analysis was performed on the remaining 32 items (Table 1). Again, the analysis identified six subscales with an eigenvalue >1, explaining 61.0% of the variance, with items loading on to the same subscales as previously. Internal reliability was good with Cronbach's alpha values between 0.80 and 0.90 for all subscales, except ‘future concerns’ (α = 0.63). Item‐total correlations were >0.3.

**Table 1:** Principal components analysis with Varimax rotation (32 items)

Item no	Item description (abbreviated)	Component					
		1	2	3	4	5	6
3	Appeared to be making a fuss about dietary needs	**0.75**	*0.21*	*0.16*	*0.15*	*0.24*	*0.05*
5	Difficult to mention misunderstanding	**0.73**	*0.14*	*0.23*	*0.06*	*0.23*	*0.01*
37	Felt you were a nuisance	**0.67**	*0.23*	*0.18*	*0.35*	*0.00*	*0.18*
39	Felt guilty about others buying gluten‐free food	**0.63**	*0.28*	*0.12*	*0.16*	*−0.04*	*0.20*
6	Received unwanted attention	**0.61**	*0.06*	*0.12*	*0.15*	*0.07*	*0.12*
4	Others misunderstood coeliac disease or dietary needs	**0.60**	*0.18*	*0.27*	*0.05*	*0.34*	*−0.11*
32	Felt uncomfortable refusing food or drink	**0.59**	*0.15*	*0.23*	*0.30*	*0.20*	*0.11*
7	Felt guilty about impact on friends and family	**0.52**	*0.23*	*0.17*	*0.34*	*0.09*	*0.29*
49	Frustrated by choice of suitable food	*0.06*	**0.76**	*0.07*	*0.06*	*0.28*	*0.04*
48	Difficulty finding food when out of the house	*0.29*	**0.67**	*0.15*	*0.18*	*0.29*	*−0.07*
42	Difficulty finding suitable food	*0.14*	**0.63**	*0.22*	*0.18*	*0.17*	*−0.16*
47	Burdened by the time to find or make gluten‐free food	*0.31*	**0.63**	*0.06*	*0.24*	*0.06*	*0.21*
50	Frustrated by planning ahead	*0.32*	**0.62**	*0.07*	*0.13*	*0.37*	*0.10*
45	Disappointed with the taste or texture of gluten‐free food	*0.06*	**0.60**	*0.15*	*0.03*	*−0.21*	*0.29*
40	Felt annoyed about the cost of gluten‐free food	*0.18*	**0.57**	*0.14*	*0.00*	*0.14*	*0.24*
44	Craved food or drinks containing gluten	*0.12*	**0.51**	*0.15*	*0.26*	*−0.29*	*0.07*
12	Had abdominal bloating	*0.21*	*0.12*	**0.74**	*0.10*	*0.02*	*0.10*
14	Had pain	*0.23*	*0.13*	**0.74**	*0.11*	*0.12*	*0.07*
11	Bothered by bowel movements	*0.13*	*0.13*	**0.69**	*0.05*	*0.12*	*0.29*
13	Had nausea or vomiting	*0.18*	*0.09*	**0.66**	*0.11*	*0.15*	*−0.06*
15	Had tiredness or a lack of energy	*0.14*	*0.19*	**0.62**	*0.35*	*0.09*	*0.14*
27	Avoided social activities	*0.22*	*0.10*	*0.19*	**0.76**	*0.22*	*0.06*
22	Felt isolated from others	*0.35*	*0.11*	*0.04*	**0.69**	*0.10*	*0.20*
29	Avoided going out to eat	*0.22*	*0.28*	*0.18*	**0.62**	*0.34*	*0.05*
36	Felt down or in low spirits	*0.31*	*0.21*	*0.36*	**0.54**	*−0.14*	*0.09*
16	Daily activities were limited by coeliac disease	*0.05*	*0.15*	*0.49*	**0.51**	*0.18*	*0.07*
31	Concerned about cross‐contamination	*0.31*	*0.22*	*0.20*	*0.27*	**0.62**	*0.32*
30	Worried about accidentally consuming gluten	*0.30*	*0.29*	*0.20*	*0.23*	**0.61**	*0.26*
1	Worried about becoming ill after eating food prepared by others	*0.40*	*0.19*	*0.36*	*0.19*	**0.55**	*0.13*
20	Worried about becoming ill when not at home	*0.22*	*0.16*	*0.36*	*0.32*	**0.51**	*0.18*
8	Worried a family member could develop coeliac disease	*0.18*	*0.12*	*0.12*	*0.13*	*0.20*	**0.72**
9	Concern about developing a related health problem	*0.19*	*0.20*	*0.35*	*0.22*	*0.18*	**0.57**

Values <0.40 are shown in italics. The highest loading for each item is shown in bold.

Components: (1) stigma, (2) dietary burden, (3) symptoms, (4) social isolation, (5) worries and concerns, and (6) future concerns. In the final CDAQ, components 5 and 6 were combined.

NB. Abbreviated CDAQ items are presented in this table. A full sample copy is available at https://innovation.ox.ac.uk/outcome-measures/coeliac-disease-assessment-questionnaire-cdaq/

As well as having low internal reliability (α = 0.63), the “future concerns” component comprised only two items. Ideally, each factor should comprise at least three items^22^ to create a reliable scale. As removal of these items would limit content validity, and as the items were conceptually similar to items in the ‘worries and concerns’ subscale, the components were combined. Internal reliability for the combined scale was good (α = 0.85) and item‐total correlations sufficient (>0.3) (Table 2).

**Table 2:** Item‐total correlations and Cronbach's alpha statistics for the final subscales

Item no		Corrected Item‐Total Correlation	Cronbach's alpha
	**Stigma (8 items)**		**0.88**
3	Appeared to be making a fuss about dietary needs	0.76	
37	Felt you were a nuisance	0.72	
5	Difficult to mention misunderstanding	0.68	
32	Felt uncomfortable refusing food or drink	0.67	
7	Felt guilty about impact on friends and family	0.63	
39	Felt guilty about others buying gluten‐free food	0.61	
4	Others misunderstood coeliac disease or dietary needs	0.59	
6	Received unwanted attention	0.52	
	**Dietary burden (8 items)**		**0.83**
48	Difficulty finding food when out of the house	0.69	
49	Frustrated by choice of suitable food	0.65	
47	Burdened by the time to find or make gluten‐free food	0.65	
50	Frustrated by planning ahead	0.65	
42	Difficulty finding suitable food	0.57	
40	Felt annoyed about the cost of gluten‐free food	0.52	
45	Disappointed with the taste or texture of gluten‐free food	0.46	
44	Craved food or drinks containing gluten	0.40	
	**Symptoms (5 items)**		**0.82**
14	Had pain	0.68	
12	Had abdominal bloating	0.67	
11	Bothered by bowel movements	0.61	
15	Had tiredness or a lack of energy	0.58	
13	Had nausea or vomiting	0.57	
	**Social isolation (5 items)**		**0.82**
27	Avoided social activities	0.71	
29	Avoided going out to eat	0.65	
22	Felt isolated from others	0.63	
36	Felt down or in low spirits	0.55	
16	Daily activities were limited by coeliac disease	0.53	
	**Worries and Concerns (6 items)**		**0.85**
31	Concerned about cross‐contamination	0.76	
30	Worried about accidentally consuming gluten	0.73	
1	Worried about becoming ill after eating food prepared by others	0.69	
20	Worried about becoming ill when not at home	0.63	
9	Concern about developing a related health problem	0.57	
8	Worried a family member could develop coeliac disease	0.47	

NB. Abbreviated CDAQ items are presented in this table. A full sample copy is available at https://innovation.ox.ac.uk/outcome-measures/coeliac-disease-assessment-questionnaire-cdaq/

#### 3.2.3 CDAQ overall score

A higher order factor analysis of the five CDAQ dimensions identified one factor with an eigenvalue >1, explaining 68.0% of the variance, indicating that it is appropriate to combine the dimension scores to create an overall index score. The subscale and overall index scores range from 0 to 100, where 0 indicates poorest quality of life and 100 indicates highest quality of life.

### 3.3 Stage 4—Assessing the reliability and validity of the CDAQ

Survey 2 achieved a 34.5% (n = 276) response rate. Eight questionnaires were excluded as the respondents did not report receiving their diagnosis from a doctor, thus leaving 268 respondents in the analysis. The majority of respondents were female (n = 166, 61.9%), married (n = 159, 59.3%), and White British (n = 225, 84.0%). The mean age of respondents was 49.5 years (SD 18.9) and the mean duration since diagnosis was 7.49 years (SD 9.67). CDAQ and SF‐36v2 scores are given in Table 3. Missing data for CDAQ items were very low, with the maximum amount of missing data for any one item being 1.1% (n = 3), which meets the PROMs quality criteria set out by Terwee et al.^26^ and indicates that the CDAQ is acceptable to adults with coeliac disease.

**Table 3:** Health‐related quality of life in coeliac disease—CDAQ and SF‐36v2 scores

	n	Mean	SD
**CDAQ**			
Overall index score	254	53.56	18.05
Stigma	262	52.58	21.55
Dietary burden	262	39.50	19.19
Symptoms	264	59.38	24.09
Social isolation	263	67.41	23.17
Worries and concerns	264	50.00	21.11
**SF‐36v2** a			
Physical Component Summary Score (PCS)	265	49.46	9.23
Mental Component Summary Score (MCS)	265	47.11	10.71
Physical Functioning (PF)	267	50.84	9.30
Role‐Physical (RP)	266	48.89	9.84
Bodily Pain (BP)	265	48.62	10.32
General Health (GH)	267	46.41	11.63
Vitality (VT)	266	47.03	10.85
Social Functioning (SF)	266	48.20	10.23
Role‐Emotional (RE)	265	48.08	10.57
Mental Health (MH)	266	47.81	10.21

a Norm‐based T scores based on 2009 US general population norms (mean 50, SD 10).

#### 3.3.1 Internal consistency reliability

Internal consistency was assessed for each CDAQ subscale. Cronbach's alpha values were: stigma (0.87), dietary burden (0.87), symptoms (0.86), social isolation (0.86) and worries and concerns (0.82), indicating good internal consistency reliability.

#### 3.3.2 Test‐retest reliability

A total of 167 respondents completed a follow‐up questionnaire, of which four were removed as they did not confirm a medical diagnosis of coeliac disease. The mean CDAQ overall index score for those returning a follow‐up questionnaire (55.87, SD 17.42) was statistically significantly higher (ie, better health‐related quality of life) than those who did not return a follow‐up questionnaire (49.87, SD 18.50), *P* = 0.010. The mean test‐retest interval was 18.93 days (range 13‐43 days).

The majority of respondents (n = 145, 89.0%) rated the impact of their coeliac disease as ‘about the same’ as when they had completed the first questionnaire and were included in the analysis. The ICCs for the CDAQ subscales were: stigma (0.85), dietary burden (0.83), symptoms (0.80), social isolation (0.87), worries and concerns (0.79) and overall index score (0.89). All were statistically significant (*P* < 0.001) indicating that the CDAQ scores are stable over time when participants report no changes (see Supporting Information for CDAQ scores).

#### 3.3.3 Convergent and divergent validity

Correlations between CDAQ dimensions and the SF‐36v2 are shown in Table 4. All correlations were in the expected direction (ie, positive). As expected, the CDAQ overall index score and subscale scores were more strongly correlated with the Mental Component Summary score than the Physical Component Summary score, with the exception of the symptoms subscale, which was strongly correlated with both (*r*_s_ ≥ 0.4, *P* < 0.001). The CDAQ symptoms subscale was also strongly correlated with Mental Health and Social Functioning. Increased symptoms would reasonably be expected to coincide with poor psychological well‐being and limited social functioning, and may explain the stronger than expected correlation between the symptoms subscale and the Mental Component Summary score.

**Table 4:** Spearman correlation coefficients between CDAQ subscales and the SF‐36v2

CDAQ Dimensions	SF‐36v2 Components		SF‐36v2 Dimensions							
	PCS	MCS	PF	RP	BP	GH	VT	SF	RE	MH
Overall index score	0.38*	0.60*	0.22*	0.34*	0.48*	0.60*	0.59*	0.58*	0.37*	0.60*
Stigma	0.18**	0.50*	0.06	0.20**	0.27*	0.41*	0.45*	0.46*	0.26*	0.49*
Dietary burden	0.31*	0.46*	0.22*	0.29*	0.36*	0.47*	0.42*	0.42*	0.33*	0.48*
Symptoms	0.42*	0.49*	0.21*	0.33*	0.51*	0.55*	0.54*	0.50*	0.31*	0.48*
Social isolation	0.35*	0.63*	0.28*	0.35*	0.44*	0.61*	0.59*	0.61*	0.44*	0.63*
Worries and concerns	0.30*	0.42*	0.15***	0.29*	0.37*	0.45*	0.43*	0.40*	0.27*	0.42*

SF‐36v2 dimensions: PCS, Physical Component Summary; MCS, Mental Component Summary; PF, physical functioning; RP, role‐physical; BP, bodily pain; GH, general health; VT, vitality; SF, social functioning; RE, role‐emotional; MH, mental health.

*P < 0.001; **P < 0.01; ***P < 0.05.

As hypothesised, moderate to strong correlations (*r*_s_ ≥ 0.4, *P* < 0.001) were found between CDAQ worries and concerns and General Health, and CDAQ social isolation and Vitality. Moderate to strong correlations (*r*_s_ ≥ 0.4, *P* < 0.001) were also found between all other CDAQ dimensions and General Health and Vitality. It is not unreasonable to expect those with poorer general health and lower energy levels to have poorer coeliac disease‐related health‐related quality of life (eg, those with increased symptoms and who find the diet more burdensome have lower energy and poorer perceptions of their health).

All other correlations were as hypothesised, although some correlations between CDAQ dimensions and Role‐Physical were marginally higher than anticipated (*r*_s_ ≤ 0.35 as opposed to *r*_s_ ≤ 0.30).

#### 3.3.4 Discriminative (known groups) validity

The mean CDAQ overall index score for men (60.91, SD = 16.81, n = 93) was 11.73 higher than women (49.18, SD = 17.36, n = 158), indicating that men report better health‐related quality of life. This difference was statistically significant (95% CI, 7.32‐16.15, *t*(249) = 5.232, *P* < 0.001).

The CDAQ overall index scores decreased from the “no impact” (72.21 ± 16.32) through “mild impact” (62.59 ± 14.38), “moderate impact” (51.57 ± 14.93), “severe impact” (40.92 ± 13.34), to “very severe impact” (28.02 ± 13.53) groups. CDAQ overall index scores were significantly different between these groups (*F*(4, 248) = 34.70, *P* < 0.001). The decrease in CDAQ overall index scores were all significant (*P* < 0.05) with the exception of the “no impact” to “mild impact” (*P* = 0.061) groups (further details can be found in the Supporting Information).

## 4 DISCUSSION

The aim of this research was to refine and reduce the number of candidate items, determine subscales, and assess the reliability and validity of the CDAQ. The final CDAQ has 32 items across five subscales: stigma (eight items), dietary burden (eight items), symptoms (five items), social isolation (five items) and worries and concerns (six items) and captures all aspects of quality of life of importance to adults with coeliac disease as identified in the qualitative phase.^18^ The CDAQ has been shown to be a reliable and valid measure.

The methods used to develop the CDAQ have been shown to be effective in the development of similar questionnaires in the past^35^, ^36^ and are compliant with best practice guidance on the development of PROMs, such as guidance provided by the US Food and Drug Administration^9^ and the International Society for Pharmacoeconomics and Outcomes Research (ISPOR).^37^ Involving potential respondents in the refinement of questionnaire items through cognitive interviews enhanced content validity, which is considered one of the most important measurement properties of PROMs.^26^ Consultation with experts can help to ensure that the content of newly developed PROMs is relevant and comprehensive^38^ and the translatability assessment helped improve the CDAQ's language and grammar and ensured that future translation issues are minimised, which is important for PROMs.^39^

A 4‐week recall period was chosen as longer recall periods are more appropriate when assessing disease‐specific health‐related quality of life, whereas shorter periods could underestimate the impact of the condition.^40^ The qualitative interviews have highlighted how the impact of coeliac disease can fluctuate according to periods of gluten consumption and the social activities undertaken. Asking patients to average symptoms over longer periods of time may provide good estimates of health status in conditions where symptoms come and go.^40^

The final CDAQ subscales represent a modification to the conceptual framework developed from the qualitative interviews (encompassing six main themes: emotional health, gluten‐free diet, relationships, impact on activities, symptoms and financial issues).^18^ However, the themes from the qualitative interviews remain represented, albeit in a potentially less obvious way. For example, financial issues identified as a qualitative theme did not become a subscale in their own right, but an item on the cost of gluten‐free food fit with the subscale of ‘dietary burden’. The impact of coeliac disease on travel and holidays was also commonly reported in the qualitative interviews, but was difficult to include as an item because the majority of respondents are unlikely to have been on holiday in the 4 week time frame covered by the CDAQ. The concept is still covered indirectly in an item on difficulties experienced with finding suitable food away from home.

Following a gluten‐free diet is known to be burdensome^2^ and the results of this study found dietary burden to have the greatest impact on health‐related quality of life. Therefore, it is essential that items addressing this burden are included in coeliac‐specific PROMs that aim to comprehensively assess health‐related quality of life. While items assessing dietary burden will be appropriate to the majority of people with coeliac disease, they may be less relevant to those who are newly diagnosed, but yet to be treated (ie, not yet following a gluten‐free diet). Further research is required to assess the reliability and validity of the CDAQ within this patient group.

This study also provides evidence that the CDAQ is a reliable and valid measure for assessing health‐related quality of life in adults with coeliac disease. Across both surveys, the internal consistency of all five subscales was within the ideal range (0.7‐0.95).^26^ Other coeliac‐specific measures have also been found to be internally consistent such as the CDQ^13^, ^41^, ^42^, ^43^, ^44^ and the CD‐QOL.^14^, ^45^ However, despite some involvement of people with coeliac disease in the development, the CDQ has not been developed on the basis of in‐depth interviews or focus groups, which are the recommended approach by the US Food and Drug Administration.^9^

Test‐retest reliability was assessed to examine the stability of test scores over time. ICCs ranged from 0.79 to 0.89, indicating that the measure is reliable. When assessed against Terwee et al's^26^ quality criteria, the CDAQ received a positive rating. The results indicate that the test‐retest reliability of the CDAQ appears superior to the CD‐QOL, for the Cohen's Kappa coefficient was not sufficient (0.63),^45^ and at least comparable with the CDQ, for which some evidence of sufficient test‐retest reliability was found.^13^, ^41^, ^42^, ^43^ However, the quality of some of the studies for both measures was poor with small sample sizes or insufficient detail about study design.

Overall, correlations between the CDAQ and SF‐36v2 were as expected, with dimensions of the CDAQ correlating more strongly with mental health dimensions of the SF‐36v2 and the Mental Component Summary score.

A possible limitation is that all survey respondents are members of Coeliac UK, although there is no evidence to suggest that health‐related quality of life in this population differs from that of the wider population of adults with coeliac disease. The cognitive interviews included participants who were not recruited through their membership of Coeliac UK. In addition, Survey 1 achieved a diverse sample (eg, 12.1% belonged to black and minority ethnic groups), which increases the likelihood that the final CDAQ will be of relevance to adults from all demographic groups. The response rate to Survey 2 (34.5%) was lower than that achieved in Survey 1 (52.0%), and is likely to be accounted for by its much greater length (as survey 2 also collected data on experiences of health care which will be reported elsewhere). Despite this, the response rate was similar to other Coeliac UK surveys.^46^ Furthermore, participants were recruited on the basis of self‐reporting their diagnosis and while they considered themselves to have been given a diagnosis of coeliac disease, it is possible that they may not have coeliac disease.

With clinical trials to develop treatments other than the gluten‐free diet underway, it is important that valid and well‐developed PROMs are available as potential endpoints for trials.^7^ In addition to symptoms and histological improvement, health‐related quality of life is regarded as a key outcome in assessing new therapeutic treatments and should be considered as a critical end point in relevant clinical trials.^17^ The results of this study further highlight the need to include measures of health‐related quality of life in clinical trials of treatments for coeliac disease, as the greatest impact on health‐related quality of life was the burden of managing a gluten‐free diet. As it is likely that the diet will remain relevant in combination with any treatments under development,^8^ it is important to have available PROMs that assess coeliac‐specific quality of life more broadly than symptoms alone. Furthermore, the CDAQ is suitable for use in clinical practice and research more broadly. Further research is underway to evaluate the CDAQ's responsiveness to change.

## AUTHORSHIP

*Guarantor of the article*: MP.

*Author contributions:* All authors contributed to the conception and design of the study. HC collected the data and led the data analysis, supported by MP and CJ. All authors contributed to the development of the Coeliac Disease Assessment Questionnaire.

All authors were involved in the writing of the manuscript and approved the final version.

## LICENCE

The Coeliac Disease Assessment Questionnaire (CDAQ) is owned and licenced by Oxford University Innovations Limited. All use of the CDAQ should be under licence which can be requested through the Clinical Outcomes team at Oxford University Innovation—https://innovation.ox.ac.uk/clinical‐outcomes/.
