# Supplementary material for: Quality of life in coeliac disease: item reduction, scale development and psychometric evaluation of the Coeliac Disease Assessment Questionnaire (CDAQ)
Source: Aliment Pharmacol Ther. 2018 Aug 20;48(8):852–62. doi: 10.1111/apt.14942 (PMC6220785; doi:10.1111/apt.14942)
Supplement: Supplementary file 1 [file APT-48-852-s001.docx]

Supporting Information

Methods

Stage 2 – Refining and pre-testing the candidate items

Cognitive interviews

Cognitive interview participants were recruited from a list of people who had expressed an interest in participating in Stage 1 (i.e. qualitative interviews), but were not interviewed, and through online social networking groups (including Facebook and Yahoo groups). The interviews were predominantly conducted over the telephone (two were face-to-face) and were audio-recorded.

Translatability assessment

As part of the translatability assessment, PharmaQuest developed a concept elaboration document which provides an in-depth description of the questionnaire and its items. This document was reviewed by HC and sent to in-country translators, together with the English version of the CDAQ. The translators were selected to represent the following language and country combinations: German (Germany), French (France), Spanish (Argentina), Arabic (Egypt), Finnish (Finland), and Simplified Chinese (China).

Stage 3 – Item reduction and scale development

In relation to the development of PROMs, a sample size of 7 respondents per item has been suggested, with a minimum of 100 respondents ^1^. Following this criterion, a minimum sample size of 357 respondents was required for Survey 1 (i.e. 7*51 items). It was estimated that a 45% response rate would be achieved, and hence 800 Members of Coeliac UK needed to be invited.

Coeliac UK’s membership is skewed towards a more elderly population, contains fewer people from black and minority ethnic (BME) groups, and more women than men. Hence, a stratified sampling strategy was adopted (Table 1).

**Table 1. Sampling stratum for Surveys 1 and 2**

| **Stratum** | **Ethnicity** | **Age group** | **Gender** | **Proposed (n)** | **Survey 1 Actual (n)** | **Survey 2 Actual (n)** |
| --- | --- | --- | --- | --- | --- | --- |
| 1 | White | <= 40 | Female | 160 | 160 | 160 |
| 2 | White | <= 40 | Male | 160 | 160 | 160 |
| 3 | White | > 40 | Female | 160 | 160 | 160 |
| 4 | White | > 40 | Male | 160 | 160 | 160 |
| 5 | BME^a^ | <= 40 | Female | 40 | 48 | 44 |
| 6 | BME | <= 40 | Male | 40 | 24 | 31 |
| 7 | BME | > 40 | Female | 40 | 40 | 40 |
| 8 | BME | > 40 | Male | 40 | 48 | 45 |
|  |  |  | **Total** | **800** | **800** | **800** |

^a^Black and Minority Ethnic (BME) Groups

Questionnaire packs were mailed from Coeliac UK and returned to the research team. Each pack included a personalised letter of invitation, participant information sheet, the questionnaire, and a pre-paid return envelope.

Analysis of Survey 1 data

To check the accuracy of data entry, 20% of questionnaires were entered twice and discrepancies (n=7, 0.10% of data entered) resolved prior to analysis.

Six candidate CDAQ items had a ‘not applicable’ option which respondents could select if they felt the item was not relevant to them.

A Principal Components Analysis (PCA) with Varimax rotation was used to identify the structure of the constructs. During the Principal Components Analysis, items were deleted one at a time, with the analysis and assessment of internal reliability performed after each item deletion, thus allowing the impact of the item’s removal on the factor structure and reliability of the scales to be assessed.

A higher order factor analysis was conducted to determine the appropriateness of combining dimension scores to create an overall score. An oblique solution was sought and hence principal axis factoring with direct oblimin rotation was used.

Stage 4 – Assessing the reliability and validity of the CDAQ

Survey 2 baseline questionnaires were mailed from Coeliac UK and returned to the research team. Follow-up questionnaires to assess test-retest reliability were sent with a covering letter and a pre-paid return envelope to all respondents who provided their contact details. Follow-up questionnaires were sent by and returned to the research team.

**Test-retest reliability - analysis**

Test-retest reliability was evaluated using the intraclass correlation coefficient (ICC), for which ICC_agreement_ with two-way random effects model was used.

Convergent and divergent validity

Table 2: Expected correlations between CDAQ subscales and SF-36v2 dimensions

| **CDAQ** | **SF-36v2** | **Expected strength of correlation** |
| --- | --- | --- |
| Stigma  Dietary burden  Social isolation  Worries and concerns | Social functioning (SF)  Mental health (MH)  MCS score | Moderate to strong (r_s_≥0.4) |
| Worries and concerns | General health (GH) | Moderate to strong (r_s_≥0.4) |
| Social isolation | Vitality (VT) | Moderate to strong (r_s_≥0.4) |
| Symptoms | Bodily pain (BP)  Vitality (VT)  PCS score | Moderate to strong (r_s_≥0.4) |
| All subscales | Physical functioning (PF)  Role-physical (RP) | Weak to moderate (r_s_≤0.3) |

Results

Stage 3 - Item reduction and scale development

Table 3: Survey 1 participants’ characteristics (n=411)

| Characteristic | | *n* | *%* |
| --- | --- | --- | --- |
| Age (years)^a^ | | 49.8±18.9 |  |
|  | 18-29 | 69 | 16.8 |
|  | 30-39 | 101 | 24.6 |
|  | 40-49 | 40 | 9.7 |
|  | 50-59 | 49 | 11.9 |
|  | 60-69 | 63 | 15.3 |
|  | 70-79 | 70 | 17.0 |
|  | 80-89 | 19 | 4.6 |
| Sex | |  |  |
|  | Female | 225 | 54.7 |
|  | Male | 186 | 45.3 |
| Marital status | |  |  |
|  | Married / civil partnership | 270 | 65.7 |
|  | Single | 98 | 23.8 |
|  | Widowed | 21 | 5.1 |
|  | Divorced | 16 | 3.9 |
|  | Separated | 6 | 1.5 |
| Ethnicity | |  |  |
|  | White British | 348 | 84.7 |
|  | Asian / Asian British | 39 | 9.5 |
|  | White Other | 13 | 3.1 |
|  | Mixed / Multiple ethnic groups | 9 | 2.3 |
|  | Black / African / Caribbean / Black British | 1 | 0.2 |
|  | Other ethnic group | 1 | 0.2 |
| Occupational status | |  |  |
|  | Full-time paid work | 169 | 41.1 |
|  | Fully retired | 117 | 28.5 |
|  | Part-time paid work | 44 | 10.7 |
|  | Doing something else | 19 | 4.6 |
|  | Looking after the home | 18 | 4.4 |
|  | Full-time education | 14 | 3.4 |
|  | Permanently sick or disabled | 10 | 2.4 |
|  | Unemployed | 8 | 1.9 |
| **Duration since diagnosis** (years)^a^ | | **8.2±10.5** |  |
|  | <1 | 47 | 11.4 |
|  | 1-4 | 139 | 33.8 |
|  | 5-9 | 96 | 23.4 |
|  | 10-14 | 52 | 12.7 |
|  | 15-19 | 21 | 5.1 |
|  | 20-24 | 10 | 2.4 |
|  | ≥25 | 39 | 9.5 |

Table 3 (continued): Survey 1 participants’ characteristics (n=411)

| Characteristic | | *n* | *%* |
| --- | --- | --- | --- |
| **Impact of coeliac disease on health** | |  |  |
|  | No impact | 39 | 9.5 |
|  | Mild impact | 119 | 29.0 |
|  | Moderate impact | 168 | 40.9 |
|  | Severe impact | 66 | 16.1 |
|  | Very severe impact | 12 | 2.9 |
| **Purposeful gluten consumption in last 12 months** | |  |  |
|  | Never | 278 | 67.6 |
|  | Rarely | 68 | 16.5 |
|  | Sometimes | 44 | 10.7 |
|  | Often | 16 | 3.9 |
|  | Always | 1 | 0.2 |
| **Diagnosis** | |  |  |
|  | Had endoscopy | 373 | 90.8 |
|  | Had blood test | 318 | 77.4 |
|  | Had other tests | 25 | 6.1 |

1. Mean±SD.

Missing data and the ‘not applicable’ response

Missing data (i.e. the proportion of non-response) was minimal, ranging from 0% to 1.2%. When including those selecting a ‘not applicable’ response option as missing data, the figures increased to between 0% and 15.5% per item, with five items missing more than 5%.

Subscale development

Following the principal components analysis (conducted on the 40 retained candidate items), eight items were removed: four items were removed due to high inter-item correlations with other items within the same subscale; one due a poor conceptual fit with other items within its component; one as it was addressing a similar issue to other items; and two items were removed due to weak loadings (<0.5) as well as being close to meeting the criteria for a floor effect (49.9% and 46.7%).

Stage 4 – Assessing the reliability and validity of the CDAQ

Of those returning a Survey 2 questionnaire, a total of 78% (n=215) consented to receive a follow-up questionnaire. The follow-up questionnaire achieved a response rate of 77.7% (n=167). Demographic and disease characteristics of Survey 2 respondents can be found in Table 4.

**Table 4. Characteristics of Survey 2 respondents**

|  | | ***Questionnaire 1*** | | ***Follow-up questionnaire*** | |
| --- | --- | --- | --- | --- | --- |
| **Characteristic** | | ***n*** | ***%*** | ***n*** | ***%*** |
| **Age (**years**)** | |  |  |  |  |
|  | 18-29 | 45 | 16.8 | 21 | 12.9 |
|  | 30-39 | 59 | 22.0 | 39 | 23.9 |
|  | 40-49 | 30 | 11.2 | 17 | 10.4 |
|  | 50-59 | 38 | 14.2 | 23 | 14.1 |
|  | 60-69 | 33 | 12.3 | 21 | 12.9 |
|  | 70-79 | 47 | 17.5 | 34 | 20.9 |
|  | 80-89 | 11 | 4.1 | 7 | 4.3 |
| **Sex** | |  |  |  |  |
|  | Female | 166 | 61.9 | 103 | 63.2 |
|  | Male | 97 | 36.2 | 59 | 36.2 |
| **Marital status** | |  |  |  |  |
|  | Married / civil partnership | 159 | 59.3 | 101 | 62.0 |
|  | Single | 75 | 28.0 | 43 | 26.4 |
|  | Widowed | 13 | 4.9 | 9 | 5.5 |
|  | Divorced | 11 | 4.1 | 6 | 3.7 |
|  | Separated | 5 | 1.9 | 3 | 1.8 |
| **Ethnicity** | |  |  |  |  |
|  | White British | 225 | 84.0 | 145 | 89.0 |
|  | Asian / Asian British | 23 | 8.6 | 9 | 5.5 |
|  | White Other | 9 | 3.4 | 6 | 3.7 |
|  | Mixed / Multiple ethnic groups | 5 | 1.8 | 1 | 0.6 |
|  | Other ethnic group | 1 | 0.4 | 1 | 0.6 |
| **Occupational status** | |  |  |  |  |
|  | Full-time paid work | 92 | 34.3 | 56 | 34.4 |
|  | Fully retired | 62 | 23.1 | 44 | 27.0 |
|  | Part-time paid work | 34 | 12.7 | 24 | 14.7 |
|  | Self-employed | 22 | 8.2 | 9 | 5.5 |
|  | Looking after the home | 14 | 5.2 | 9 | 5.5 |
|  | Full-time education | 12 | 4.5 | 5 | 3.1 |
|  | Doing something else | 7 | 2.6 | 5 | 3.1 |
|  | Permanently sick or disabled | 7 | 2.6 | 1 | 0.6 |
|  | Unemployed | 4 | 1.5 | 4 | 2.5 |
|  | Missing / multiple data | 14 | 5.2 | 2 | 1.2 |

**Table 4 (continued). Characteristics of Survey 2 respondents**

|  | | | ***Questionnaire 1*** | | | | ***Follow-up questionnaire*** | | | | |  |
| --- | --- | --- | --- | --- | --- | --- | --- | --- | --- | --- | --- | --- |
| **Characteristic** | | | ***n*** | | ***%*** | | ***n*** | ***%*** | | | |  |
| **Duration since diagnosis** (years) | |  | |  | |  | | | |  | | |
|  | <1 | 28 | | 10.4 | | 12 | | | | 7.4 | | |
|  | 1-4 | 113 | | 42.2 | | 69 | | | | 42.3 | | |
|  | 5-9 | 40 | | 14.9 | | 29 | | | | 17.8 | | |
|  | 10-14 | 40 | | 14.9 | | 28 | | | | 17.2 | | |
|  | 15-19 | 15 | | 5.6 | | 9 | | | | 5.5 | | |
|  | 20-24 | 4 | | 1.5 | | 3 | | | | 1.8 | | |
|  | ≥25 | 16 | | 6.0 | | 7 | | | | 4.3 | | |
| **Impact of coeliac disease on health** | |  | |  | |  | | | |  | | |
|  | No impact | | 24 | | 9.0 | | 17 | | 10.4 | |  |  |
|  | Mild impact | | 80 | | 29.9 | | 48 | | 29.4 | |  |  |
|  | Moderate impact | | 107 | | 39.9 | | 69 | | 42.3 | |  |  |
|  | Severe impact | | 38 | | 14.2 | | 21 | | 12.9 | |  |  |
|  | Very severe impact | | 17 | | 6.3 | | 6 | | 3.7 | |  |  |
| **Purposefully consumed gluten in last 12 months** | | |  | |  | |  | |  | |  |  |
|  | Never | | 193 | | 72.0 | | 115 | | 70.6 | |  |  |
|  | Rarely | | 40 | | 14.9 | | 25 | | 15.3 | |  |  |
|  | Sometimes | | 28 | | 10.4 | | 17 | | 10.4 | |  |  |
|  | Often | | 4 | | 1.5 | | 4 | | 2.5 | |  |  |
|  | Always | | 1 | | 0.4 | | 0 | | 0 | |  |  |

Note: Missing data <5% is not reported.

SF-36v2 scores

Mean SF-36v2 scores from Survey 2 are reported using the original 0-100 scoring system in Table 5.

**Table 5. Survey 2 SF-36v2 scores (0-100) (n=267)**

|  | | | ***Scores (0-100)*** | | |
| --- | --- | --- | --- | --- | --- |
|  | | ***n*** | ***Mean*** | ***SD*** | ***Range*** |
| **SF-36v2 scores** |  |  |  |  |  |
| Physical Functioning (PF) | 267 | 82.48 | 24.29 | 0 - 100 |  |
| Role-Physical (RP) | 266 | 77.00 | 27.39 | 0 - 100 |  |
| Bodily Pain (BP) | 265 | 66.82 | 25.58 | 0 - 100 |  |
| General Health (GH) | 267 | 57.76 | 24.47 | 0 - 100 |  |
| Vitality (VT) | 266 | 50.78 | 22.83 | 0 - 100 |  |
| Social Functioning (SF) | 266 | 77.21 | 25.50 | 0 - 100 |  |
| Role-Emotional (RE) | 265 | 80.63 | 25.30 | 0 - 100 |  |
| Mental Health (MH) | 266 | 69.16 | 19.51 | 5 - 100 |  |

Test-retest reliability

**Table 6. CDAQ scores for respondents completing both questionnaires whose health was unchanged, n=145**

|  | ***Survey 1*** | | | ***Survey 2*** | | |
| --- | --- | --- | --- | --- | --- | --- |
|  | ***n*** | ***Mean*** | ***SD*** | ***n*** | ***Mean*** | ***SD*** |
| Overall score | 140 | 56.15 | 17.43 | 139 | 57.07 | 18.54 |
| Stigma | 144 | 54.19 | 21.59 | 145 | 55.24 | 22.65 |
| Dietary burden | 143 | 41.24 | 19.27 | 140 | 42.83 | 20.07 |
| Symptoms | 145 | 61.72 | 22.46 | 143 | 63.46 | 23.27 |
| Social isolation | 143 | 70.28 | 21.80 | 144 | 69.48 | 22.98 |
| Worries and concerns | 144 | 53.62 | 20.65 | 144 | 55.50 | 20.56 |

Discriminative (known groups) validity

**Table 7. Comparisons of CDAQ overall index scores by self-reported impact of coeliac disease groups**

|  |  | ***CDAQ overall index score*** | | | |
| --- | --- | --- | --- | --- | --- |
| **Group (a)** | **Group (b)** | ***Mean difference between (a) and (b)*** | ***SE*** | ***95% CI*** | ***p*** |
| No impact | Mild impact | 9.62 | 3.60 | -0.28 to 19.51 | 0.061 |
| Mild impact | Moderate impact | 11.02 | 2.21 | 4.96 to 17.08 | **<0.001** |
| Moderate impact | Severe impact | 10.65 | 2.79 | 2.98 to 18.31 | **0.002** |
| Severe impact | Very severe impact | 12.90 | 4.47 | 0.63 to 25.17 | **0.034** |

References

1. Terwee CB, Bot SD, de Boer MR, et al. Quality criteria were proposed for measurement properties of health status questionnaires. *J Clin Epidemiol.* 2007;60:34-42.
